# Exploring professional nurses’ perceptions of structural, organizational, and workplace constraints affecting nursing assessment in a selected public hospital in South Africa: a qualitative study

**DOI:** 10.3389/frhs.2026.1826594

**Published:** 2026-05-13

**Authors:** G. Hlungwani, T. E. Mutshatshi, L. W. Mokhwelepa

**Affiliations:** 1 Department of Nursing Science, Faculty of Health Sciences, University of Limpopo, Sovenga, South Africa; 2 School of Medicine, Faculty of Health Science, University of Limpopo, Sovenga, South Africa

**Keywords:** health system factors, nursing assessment, organisational barriers, public hospitals, quality of care, workplace constraints

## Abstract

**Background:**

Efficient and effective nursing assessment is essential for developing comprehensive nursing care plans that support clinical decision-making and quality service delivery. However, nursing assessment is influenced by broader healthcare system conditions.

**Objective:**

This study aimed to explore nurses’ perceptions of structural, organizational, and workplace constraints affecting nursing assessment and how these constraints influence the quality of care delivery in a selected public hospital in South Africa.

**Methods:**

A qualitative and exploratory descriptive design was used. Twenty professional nurses were selected through purposive sampling based on clinical experience and direct involvement in care delivery. Data were collected through semi-structured, in-depth interviews guided by an interview schedule. Interviews were audio-recorded and supplemented with field notes. Data were transcribed verbatim and analyzed using Tesch's open coding method to generate themes and sub-themes. Ethical principles were strictly adhered to throughout the study.

**Results:**

Two main themes and six subthemes emerged, highlighting significant structural and organizational constraints within the healthcare system. These included staff shortages, overcrowding, inadequate infrastructure, limited material resources, and inefficient resource allocation systems, all of which negatively influenced nursing assessment processes. Participants described how these systemic pressures resulted in workload escalation, fatigue, burnout, and reduced capacity for timely and comprehensive assessment. To cope, professional nurses reported relying on task prioritisation, workflow adjustments, and informal system adaptations within an overstretched service environment.

**Conclusion:**

Nursing assessment in the study setting is significantly shaped by structural and organizational constraints within the healthcare system. These systemic challenges negatively affect workforce well-being and the quality and timeliness of care processes. Strengthening healthcare staffing models, infrastructure capacity, and resource allocation systems is essential to improve nursing assessment and overall service delivery.

## 1 Introduction

Effective nursing assessment is a core principle of safe and high-quality patient care. However, numerous organizational, structural, and workplace challenges compromise its effectiveness in clinical settings. Communication barriers, including language differences and low health literacy levels, often hinder accurate history taking and symptom identification during initial assessment of care (1). Moreover, increasing patient acuity and multimorbidity complicate comprehensive assessment processes, requiring advanced clinical reasoning skills that may not always be sufficiently developed (2). Similarly, heavy workloads and inadequate nurse-patient ratios reduce the time available for holistic patient assessment and evaluation (3). Interestingly, research suggests that many nurses rely more heavily on technology than on comprehensive physical examination skills, potentially weakening direct patient assessment practices (4). Consequently, gaps in clinical skills and competence, environmental pressures, and patient-related complexities collectively undermine the thoroughness and accuracy of nursing assessments (5).

Conversely, ineffective or incomplete nursing assessment has significant consequences for the quality of patient care. Missed nursing care, often resulting from insufficient assessment, has been directly associated with adverse patient outcomes such as falls, pressure injuries, and medication errors (6). Moreover, inadequate surveillance and delayed recognition of patient deterioration increase morbidity and mortality rates (7). Similarly, poor assessment practices negatively affect continuity of care and reduce patient satisfaction levels (8). Interestingly, burnout among nurses is frequently linked to workload stress, which has been shown to mediate the relationship between poor assessment environments and compromised care quality (9). Consequently, ineffective nursing assessment does not merely represent a procedural gap but rather a systemic threat to patient safety and overall healthcare quality (10).

Patient-related and systemic barriers to effective nursing assessment remain prominent within hospital settings. High nurse turnover and workforce shortages limit opportunities for thorough patient evaluation (11). Moreover, time constraints associated with increased administrative documentation reduce direct patient interaction and assessment quality (12). Similarly, complex chronic disease burdens in American hospitals require advanced assessment competencies that may not always align with staffing realities (13). Interestingly, Magnet-recognized hospitals demonstrate improved patient outcomes, suggesting that supportive practice environments strengthen assessment quality (14). Consequently, organizational support and workforce stability directly influence the effectiveness of nursing assessment and patient care outcomes (15).

Similarly, in Australia, healthcare systems face distinct yet comparable assessment challenges. In Australia, rural and remote healthcare settings experience workforce shortages that limit comprehensive nursing assessment capacity (16). Moreover, cultural and linguistic diversity among patients presents communication challenges that complicate accurate evaluation (17). In China, rapid healthcare reforms and high patient volumes contribute to workload pressures that affect clinical assessment practices (18). Interestingly, nurse education reforms in China aim to strengthen clinical reasoning and assessment skills, yet disparities remain between urban and rural institutions (19). Consequently, although Europe and China differ structurally, both contexts demonstrate how systemic constraints and patient complexity shape nursing assessment effectiveness and influence patient care quality (20).

In South Africa, these challenges are intensified within the public healthcare sector, where resource limitations and increased disease burden intersect. Studies indicate that high patient loads and staff shortages significantly impede nurses’ ability to conduct comprehensive assessments in public hospitals (21). Moreover, poor practice environments, including inadequate infrastructure and limited managerial support, negatively affect perceived care quality (22). Similarly, communication barriers arising from linguistic diversity complicate patient history taking and assessment accuracy (23). Interestingly, research has shown that nurses in South Africa often report moral distress when unable to deliver thorough care due to systemic constraints (24). Consequently, within South African public hospitals, patient-related assessment challenges compounded by structural inequities have profound implications for patient safety and quality of care, underscoring the importance of context-specific qualitative inquiry (25).

Despite growing international evidence on barriers to effective nursing assessment, there remains limited qualitative research that specifically explores nurses’ lived experiences of structural, organizational, and workplace constraints affecting nursing assessment within South African public hospital settings. Existing studies tend to focus on quantitative indicators of workload, staffing ratios, or patient outcomes, with less attention given to how nurses perceive and navigate these constraints in their daily clinical practice. Furthermore, there is a lack of context-specific evidence from the rural South African public hospital settings where resource limitations, increased burden of diseases, and high patient care demand may further shape assessment practices. This study contributes new knowledge by providing an in-depth qualitative understanding of nurses’ lived experiences of structural, organizational, and workplace constraints affecting nursing assessment in a South African public hospital.

### 1.1 Objective of the study

This study aimed to explore and describe nurses’ perceptions of structural, organisational, and workplace constraints affecting nursing assessment, and to understand how these constraints are perceived to influence the quality of care delivery in a selected public hospital in South Africa.

### 1.2 Theoretical framework

This study is guided by Donabedian's Structure–Process–Outcome (SPO) Model of healthcare quality (26). The model posits that healthcare quality is determined by the relationship between structural factors (the environment in which care is delivered), process factors (how care is provided), and outcomes (the results of care). In the context of this study, structural factors include staffing levels, resource availability, patient load, and institutional support within the selected public hospital. These structural elements influence the process of nursing assessment, including patient history taking, physical examination, monitoring, and documentation. When structural constraints compromise assessment processes, adverse care-related outcomes such as delayed clinical decision-making, procedural inefficiencies, and reduced service effectiveness may occur. The framework, therefore, provides a systematic lens through which structural, organizational, and workplace constraints and their influence on nursing assessment processes and care outcomes within a healthcare system context can be examined.

**Figure 1:**
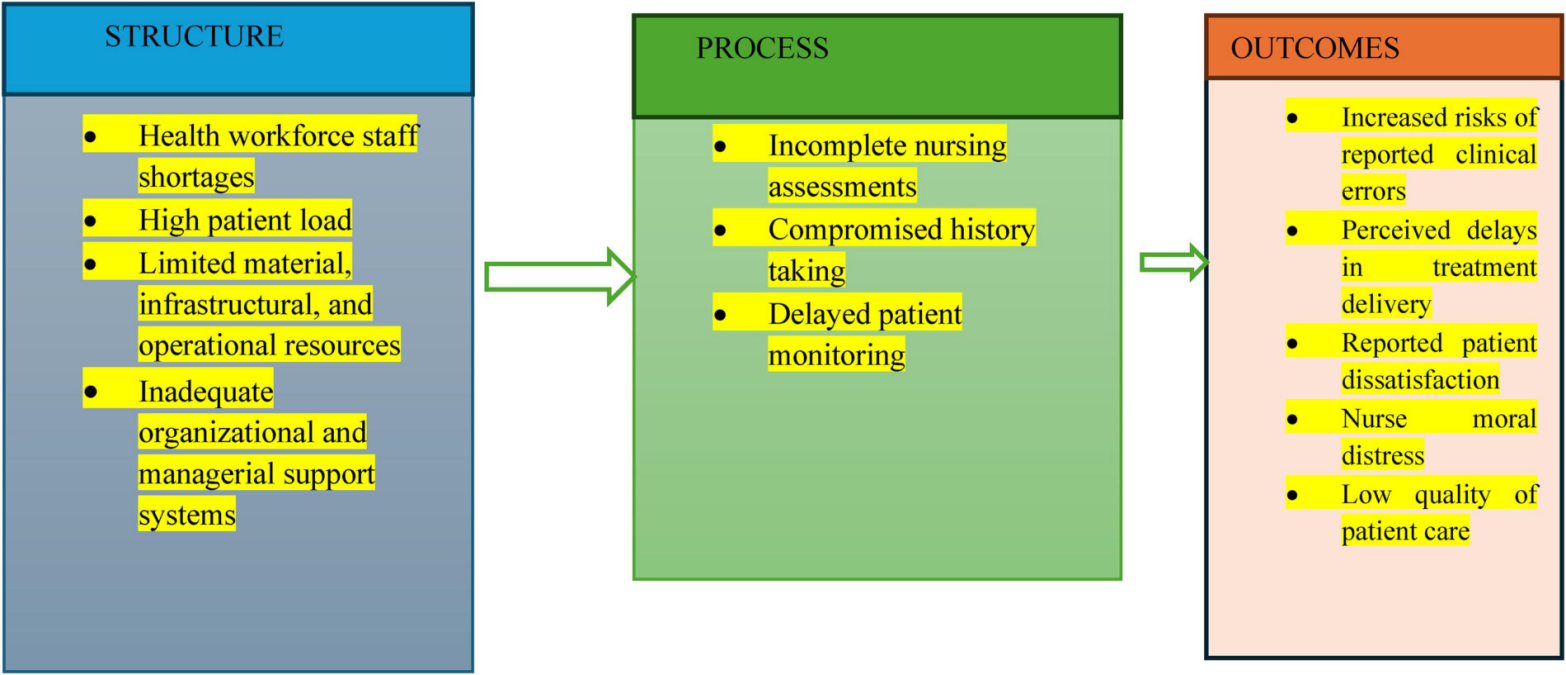
A schematic presentation of the theoretical model adapted from Donabedian (26).

The authors have conceptualized and adapted Donabedian's Structure–Process–Outcome Model to illustrate how structural and organizational workplace constraints within a public healthcare setting influence nursing assessment and subsequent care delivery outcomes in a South African public hospital. This conceptual framework highlights the relationships between structural factors, assessment processes, and care-related outcomes within a health system context, rather than attributing outcomes to patient-related factors. See Figure 1 for a visual representation.

## 2 Methodology

### 2.1 Research design

A qualitative and exploratory descriptive research design was used to explore the organizational, structural, and work-related constraints that affect effective nursing assessment and to examine how these challenges influence the quality of patient care in a selected public hospital in South Africa.

### 2.2 Research setting

This study was conducted at a selected public hospital in the Madibeng Sub-District of the North-West Province, South Africa. The hospital is located in a rural farming and mining community approximately 55 km north-west of Pretoria, the capital city of South Africa, and serves a large and diverse rural population. It is a 267-bed facility comprising various wards, including general medical, surgical, and intensive care units, from which participants were recruited. The hospital was purposefully selected, as it is considered representative of similar rural district hospitals in the North-West Province, characterized by high patient volumes, limited resources, and staffing constraints, making it an appropriate site for exploring contextual challenges in nursing practice.

### 2.3 Study participants and sampling

The study population comprised all professional nurses working in the medical, surgical, and intensive care units of a selected public hospital in the North-West Province, South Africa. A non-probability purposive sampling technique was used to select participants based on their ability to provide rich, relevant, and detailed information aligned with the study objectives.

The sampling logic was grounded in an information-rich case selection strategy, where participants were deliberately chosen because of their direct involvement in patient care and their experiential knowledge of the phenomenon under investigation. This approach ensured that the sample reflected depth rather than statistical representativeness, which is consistent with qualitative research principles. To enhance variation and comprehensiveness of perspectives, a maximum variation sampling strategy was incorporated within the purposive framework. This involved deliberately selecting nurses from different clinical units (medical, surgical, and intensive care), each of which presents distinct workloads, patient acuity levels, staffing demands, and clinical decision-making contexts. This variation was intended to capture a broad spectrum of experiences and reduce the risk of unit-specific bias in the findings.

A total of twenty (20) participants were recruited, consisting of seventeen (17) females and three (3) males. Participant selection was also guided by professional role homogeneity (all being registered professional nurses) to ensure that differences in responses were attributable to clinical context rather than differences in professional category or scope of practice. Inclusion criteria required participants to be registered professional nurses with at least 1 year of working experience in the selected hospital and actively involved in direct patient care within the identified wards. The 1-year minimum experience criterion was applied to ensure that participants had sufficient exposure to institutional workflows, clinical challenges, and ward-specific dynamics to provide meaningful and reflective accounts.

Participants were recruited across the three units using ward-based recruitment lists provided by operational managers. Within each unit, eligible nurses were identified and approached during shift intervals to ensure equitable access across day and night duty staff, thereby reducing selection bias linked to shift allocation. Recruitment continued iteratively across wards to ensure balanced representation of experiences from all three clinical environments. The participants characteristics are displayed in Table 1.

**Table 1:** Characteristics of the participants.

Participant's pseudonyms	Gender	Age	Highest qualification	Year of nursing experience	Ward/Unit	Employment type
P1	Female	34	Diploma in nursing	10	Medical ward	Permanent
P2	Female	34	Diploma in nursing	8	Medical ward	Permanent
P3	Female	33	Diploma in nursing	10	Medical ward	Permanent
P4	Female	25	Diploma in nursing	5	Medical ward	Permanent
P5	Male	25	Degree in nursing	5	Medical ward	Permanent
P6	Female	31	Diploma in nursing	7	Medical ward	Permanent
P7	Female	34	Diploma in nursing	9	Medical ward	Permanent
P8	Female	47	Diploma in nursing	20	Intensive care unit	Permanent
P9	Female	25	Degree in nursing	5	Intensive care unit	Permanent
P10	Female	29	Diploma in nursing	8	Intensive care unit	Permanent
P11	Female	50	Degree in nursing	33	Intensive care unit	Permanent
P12	Female	55	Diploma in nursing	30	Intensive care unit	Permanent
P13	Female	48	Diploma in nursing	25	Intensive care unit	Permanent
P14	Male	25	Degree in nursing	4	Intensive care unit	Permanent
P15	Female	27	Diploma in nursing	6	Surgical ward	Permanent
P16	Female	29	Diploma in nursing	8	Surgical ward	Permanent
P17	Female	30	Diploma in nursing	8	Surgical ward	Permanent
P18	Female	35	Diploma in nursing	10	Surgical ward	Permanent
P19	Male	36	Degree in nursing	8	Surgical ward	Permanent
P20	Female	27	Diploma in nursing	5	Surgical ward	Permanent

### 2.4 Data collection

In this study, data were collected from 20 July 2023 to 02 October 2023 using semi-structured, one-to-one interviews guided by an interview schedule. The interview guide was developed following an extensive review of relevant literature and aligned with the study objectives to ensure that all key domains of inquiry were adequately explored. To enhance credibility, the interview guide was reviewed by qualitative research experts and piloted with two professional nurses from a similar setting who were not part of the main study. Minor refinements were made to the interview guide to improve clarity, flow, and sequencing of questions based on the pilot findings. The findings of the pilot study were not included in the main study.

Interviews were conducted in a private, empty office within each unit to ensure confidentiality, minimize interruptions, and create a comfortable environment for participants. All applicable COVID-19 safety protocols, including hand hygiene, mask usage, and physical distancing, were strictly adhered to throughout the data collection process. The interviews were conducted in English, as all professional nurses were proficient in English as the official language of professional communication. However, participants were encouraged to express themselves in their preferred language where necessary to ensure clarity and depth of responses. Where local languages were used, the researcher translated responses into English while ensuring meaning preservation through careful checking during transcription.

Each interview lasted approximately 30–45 min. The researcher initiated the interviews using open-ended questions from the interview guide, followed by probing questions based on participants’ responses to obtain rich, in-depth information about the phenomenon under investigation. This flexible probing approach allowed participants to elaborate on their experiences and ensured depth and clarity of responses. All interviews were audio-recorded with participants’ consent, and field notes were taken to capture non-verbal cues, contextual details, and initial reflections. Data were transcribed verbatim shortly after each interview to ensure accuracy and to allow for concurrent analysis during the data collection process.

Data saturation was reached at the 20th interview. This determination was made through continuous concurrent data collection and analysis, where repeated comparison of emerging codes across interviews demonstrated increasing redundancy of information. Saturation was therefore not based on sample size alone but also on the point at which additional interviews no longer contributed new conceptual insights or altered the developing thematic structure, thereby providing a more robust justification of data adequacy.

In this study, the primary researcher was a male academic, who had been trained in qualitative processes and personally conducted participant recruitment and all interviews. His identity and positionality as a male researcher in a predominantly female nursing environment may have influenced participant responses, particularly in relation to openness and perceived power dynamics during interviews. However, efforts were made to minimize this influence by maintaining a neutral, respectful, and non-judgemental stance throughout data collection, and all preconceived ideas were kept aside during data collection to minimize bias. The researcher had no supervisory, managerial, or clinical relationship with participants, which helped to reduce coercion and support voluntary participation. It is also acknowledged that his outsider position within the clinical units may have facilitated candid responses while also requiring deliberate rapport-building to establish trust. Throughout the study, the researcher engaged in ongoing reflexive consideration of how his assumptions, identity, and positioning could influence the interpretation of the data, ensuring that data analysis remained grounded in participants’ accounts rather than researcher bias.

The following questions, based on Donabedian's SPO Model, were asked to all participants during the interview:

1. Structure (Environment & Resources)

How do staffing, resources, and your work environment affect your ability to assess patients effectively?

(1) Process (Nursing Assessment Practices)

Can you kindly describe the main challenges you face when conducting nursing assessments on admitted patients?

(1) Outcome (Impact on Patient Care)

In your experience, what happens to patient care when nursing assessments are incomplete or delayed?

Probing questions, such as “*could you please elaborate on that?*” and “*what do you mean by that?*” were employed to elicit deeper insights into the participants’ challenges experienced with nursing assessment practice in the selected public hospital.

### 2.5 Data analysis

The collected data were transcribed and analyzed using Tesch's eight-step inductive and open-coding technique (27). The primary author initiated the analysis with an in-depth review of data obtained from one-on-one semi-structured interviews to develop a comprehensive understanding of the challenges experienced by nurses during nursing assessments. The process commenced with the careful examination of a single transcript to gain a preliminary sense of the content, while making notes on initial impressions and emerging ideas. In the subsequent step, topics identified during the initial review were systematically grouped into columns labelled as main topics, unique topics, and miscellaneous items. Abbreviated codes were then assigned to each topic, and the corresponding sections within the text were marked accordingly. To ensure accuracy and completeness, the data organization was revisited to incorporate any newly emerging categories or codes. Next, the most descriptive words and phrases representative of the themes were selected and transformed into overarching categories. Subsequently, all data relevant to each identified theme were consolidated and organized into a single column to facilitate comprehensive analysis. To enhance the trustworthiness of the findings, the researcher maintained ongoing engagement with supervisors throughout the data analysis process. Additionally, an independent coder and an experienced qualitative researcher with a PhD in nursing science was engaged to independently identify themes and sub-themes. Following the independent analysis, the researcher and the coder convened to compare their findings and reach a consensus on the final themes and sub-themes. As consensus was achieved, no further recording was deemed necessary.

### 2.6 Measures to ensure trustworthiness

Measures to ensure trustworthiness were embedded throughout the analytic process, with a focus on how interpretive decisions were actively made, questioned, and refined during analysis rather than treated as procedural requirements reported after the fact (28). Instead of accepting early interpretations, initial codes and emerging ideas were repeatedly compared across transcripts to check whether they were supported across multiple participants or reflected isolated viewpoints, leading to revision, merging, or discarding of codes where inconsistencies were identified. This process ensured that the developing themes reflected patterned meaning across the dataset rather than descriptive summaries of individual accounts.

Close attention was given to maintaining a clear analytic trail between raw data and final themes, with frequent return to original transcripts during coding to verify that interpretations remained grounded in participants’ actual wording and context. In several instances, theme boundaries were adjusted when overlapping codes were identified across different interviews, prompting reclassification to ensure conceptual clarity and internal consistency.

An independent coder analysed the dataset using the same transcripts, and differences in coding were not treated as errors but as prompts for deeper discussion about meaning and interpretation. These discussions led to refinement of code definitions and clarification of thematic distinctions, particularly where similar concepts were interpreted differently (e.g., workload pressure vs. resource constraint impacts). This iterative exchange strengthened the precision and consistency of the final thematic structure.

Reflexivity was not treated as a static statement but as an ongoing analytical practice. The researcher maintained reflective notes throughout data collection and analysis, documenting moments where prior assumptions about nursing workload and assessment were challenged by participant accounts. For example, early expectations that patient-related factors would dominate the data shifted as analysis progressed, with structural and organizational constraints emerging as more central across interviews. These reflections were actively used during coding to question initial interpretations and prevent premature conclusions, ensuring that findings remained closely tied to the data.

Overall, trustworthiness was achieved through continuous analytic checking, iterative refinement of interpretations, and sustained reflexive engagement with the data, ensuring that the findings represent a carefully constructed interpretation of participants’ reported experiences within their specific clinical context.

### 2.7 Ethical considerations

Ethical clearance for the study was obtained from the Turfloop Research Ethics Committee (TREC/553/2022: UG). Additionally, authorization to conduct the research was granted by the North-West Provincial Ethical Research Committee, the Bojanala District office, and the chief executive officers and nursing management of the selected public hospital. Prior to data collection, professional nurses were thoroughly briefed and provided with comprehensive information to facilitate an informed consent and voluntary decision regarding their participation. Informed written consent was obtained after the researchers clearly articulated the study's objectives, procedures, and their own credentials to establish credibility. Professional nurses were also given the opportunity to ask questions before signing the consent forms. Participants were explicitly informed of their right to decline participation or to withdraw from the study at any time without any negative consequences. To ensure transparency and uphold participants’ autonomy, the researcher clearly explained the rationale and procedures for audio recording and note-taking during the interviews. Anonymity was rigorously maintained by keeping participants’ identities confidential and inaccessible to anyone outside the research team. To uphold confidentiality, pseudonyms were assigned during data collection, with each professional nurse identified as Participant 1, 2, or 3 during the one-on-one semi-structured interviews. The collected data was kept on a password-protected laptop to further safeguard the confidentiality of the information.

## 3 Results

**Figure 2:**
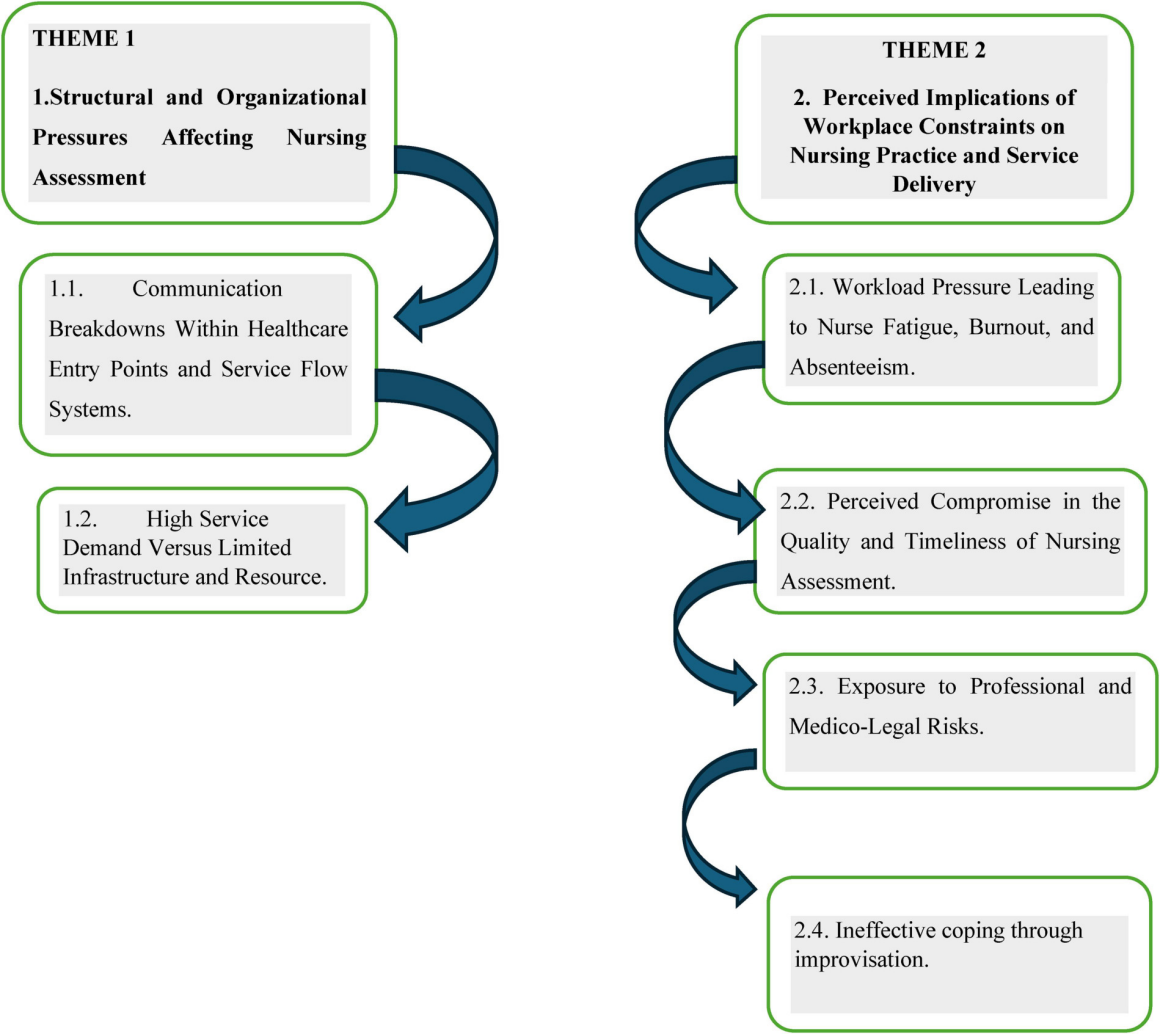
Themes and subthemes.

In this study, two themes and six subthemes surfaced, as demonstrated in Figure 2 below. The interviews were conducted among twenty (20) professional nurses who work in a hospital in the Madibeng sub-District of the North-West Province, who have a better understanding of organizational, structural, and work-related constraints when assessing patients during their nursing care.

### 3.1 Structural and organizational pressures affecting nursing assessment

This theme describes the structural, systemic, and organizational conditions within the healthcare environment that negatively influence nursing assessment practices. In alignment with Donabedian's Structure–Process–Outcome Model, these challenges are conceptualized as system-level determinants embedded within healthcare delivery structures, shaping care processes and influencing overall outcomes.

#### 3.1.1 Communication breakdowns within healthcare entry points and service flow systems

Participants highlighted inefficiencies in communication systems across healthcare entry points and service delivery areas as a major barrier to effective nursing assessment. Inconsistent messaging, fragmented service delivery pathways, and lack of standardized communication protocols between units were reported to contribute to misunderstandings and tension during assessment processes. These challenges reflect structural weaknesses in healthcare communication systems, particularly in how information is transferred across departments and service points.

As evidenced by:

“Within the service entry system, there are strong assumptions that staff are unapproachable, and this influences interactions once individuals arrive for care.” (P1)

“At times, cases arrive from entry units where care processes were not properly managed, which later creates difficulty in obtaining accurate information during assessment.” (P9)

#### 3.1.2 High service demand vs. limited infrastructure and resource capacity

Participants highlighted severe system-level workload pressures resulting from overcrowded facilities, staff shortages, insufficient bed capacity, and constrained material resources. These conditions reflected an imbalance between service demand and institutional capacity, where service delivery points were consistently operating beyond intended capacity thresholds. Nursing staff reported being required to manage excessive workloads that exceeded safe operational standards, including responsibilities that extended beyond manageable clinical limits within existing staffing configurations. In addition, infrastructure limitations such as inadequate bed capacity and restricted clinical space further intensified operational strain.

Participants further indicated that increased service demand within the catchment area was not matched by proportional adjustments in staffing levels, budget allocations, or infrastructure expansion, resulting in persistent system overload. These experiences illustrate structural inefficiencies in healthcare planning, resource allocation, and capacity management, which collectively impact the execution of nursing responsibilities, including assessment processes and care delivery.

In terms of Donabedian's Structure–Process–Outcome Model, these represent clear structural deficiencies that constrain care processes, particularly limiting the ability to conduct comprehensive assessments and maintain optimal service delivery standards.

As evidenced by:

“The main challenge is the overall workload distribution within the unit, where service demand exceeds safe staffing capacity, requiring continuous task management across a high number of cases.” (P6)

“Resource allocation has not been adjusted in line with increased service demand in the catchment area, resulting in recurring shortages of essential operational supplies and capacity constraints.” (P18)

“Operational space constraints within service areas frequently require temporary redistribution of functional clinical areas to accommodate overflow cases.” (P12)

“Staffing structures have not expanded proportionally with increased service demand, resulting in sustained workforce shortages within service units.” (P16)

### 3.2 Perceived implications of workplace constraints on nursing practice and service delivery

This theme captures the outcomes that arise when structural deficiencies disrupt nursing processes. The findings demonstrate how compromised care processes translate into negative professional and patient outcomes. These outcomes are presented as reported experiences of nurses within their specific work environment.

#### 3.2.1 Workload pressure leading to nurse fatigue, burnout, and absenteeism

Participants described persistent exhaustion due to excessive workload, leading to physical illness, emotional strain, and absenteeism. Some nurses reported skipping meals or working entire shifts without rest. Within Donabedian's framework, burnout and absenteeism represent negative outcomes stemming from structural inadequacies and overwhelming care processes. However, in the context of this study, these are understood as nurses’ perceived experiences of the effects of workload pressures rather than objectively measured outcomes.

This is explored from the following:

“I think the other cause of shortage of staff is the absenteeism of the nurses due to exhaustion (P2)”.

“I am saying this because as we overstretch ourselves, we end up getting exhaustion, and as we exhaustion we get sick and absent ourselves (P11)”.

“Sometimes you even go back home with your lunch box without eating because by the time it's lunch time there is still a lot of work that is not yet done. By that time, you will find that I am the only professional nurse in the unit “(P5)”.

#### 3.2.2 Perceived compromise in the quality and timeliness of nursing assessment

Nurses acknowledged that heavy workloads often forced them to rush assessments, omit important clinical details, or rely on junior staff for patient monitoring. Time constraints made thorough nursing assessments difficult, especially when managing multiple patients simultaneously. These accounts reflect participants’ perceptions that workload pressures influenced how nursing assessments were prioritized and conducted in practice, particularly in relation to completeness and timeliness.

According to Donabedian's model, structural shortages disrupt the process of proper assessment, leading to compromised patient outcomes, including potential missed complications and reduced quality of care. Within this study context, participants described these structural constraints as contributing to perceived challenges in carrying out comprehensive nursing assessments as intended.

As evidenced by:

“If there is a new admission, for example, I quickly come and do a nursing assessment, but mostly, I don’t do it effectively, and I miss some of the things during the assessment because there is other work waiting for me (P1)”.

“So, in conclusion, I can say that, indeed nursing assessment is not really being done effectively because when the patient is bedridden, I rely on my juniors for any abnormality that may develop from the patient, such as the development of a pressure sore (P4)”.

“Remember I am allocated 15 patients, and nursing assessment can take about 30 min, so honestly, I am unable to do so. I have a lot of responsibility, so I end up compromising some of the nursing care (P9)”.

#### 3.2.3 Exposure to professional and medico-legal risks

Participants reported delegating complex tasks due to staffing shortages and occasionally delaying or inaccurately completing documentation. These practices, as described by nurses, were driven by workload pressures and limited staffing capacity. These reflect participants’ perceptions of increased professional vulnerability arising from the demands of their working environment, particularly when required to manage responsibilities beyond available staffing support.

In the Structure–Process–Outcome model, strained processes resulting from structural limitations lead to adverse professional outcomes, including ethical and medico-legal risks. Within the context of this study, this relationship is interpreted as participants perceived exposure to such risks rather than confirmed incidents of legal or ethical violations.

As evidenced by:

“Sometimes when I am not coping, I end up delegating our enrolled nurses to nurse a ventilated patient, but I still make sure that I supervise them (P11)”.

“Sometimes documentation is delayed because we are attending to many patients at the same time, and we try to complete it later when the workload reduces” (P2).

#### 3.2.4 Ineffective coping through improvisation

To manage overwhelming demands, nurses described improvising by prioritizing patients according to severity, working with incomplete information, or adapting care practices to resource limitations. While these strategies enabled continuity of care, they reflected deviations from ideal standards. These accounts represent participants’ perceived coping strategies used in response to workload pressures and resource constraints within their clinical environment.

Within Donabedian's framework, such adaptations represent altered processes necessitated by structural inadequacies, which may further influence care outcomes. However, in this context, these implications are interpreted as perceived effects on the organization and delivery of care rather than objectively measured impacts on patient outcomes.

This is evidenced by:

“Honestly, sometimes I don’t cope, but I try to improvise, or I categorize my patients according to the severity of their conditions for that day, not saying others are not important (P8)”.

“Sometimes we try to get an interpreter, and if we can’t find any, we just work on the history that we have because there is nothing more we can do (P19)”.

“Honestly, I can’t spend such a lot of time on each patient while the ward is full of patients (p6)”.

## 4 Discussion

The structural challenges described by nurses in the findings, including high patient volumes, limited infrastructure, and staff shortages, are consistent with broader literature that reports similar constraints in many healthcare settings. Research has described how inadequate staffing and resource limitations are associated with difficulties in completing nursing tasks and conducting comprehensive assessments (29). In Malawi, for example, nurses reported that high workload undermined their ability to provide optimal care and maintain patient safety (30). Within the context of the present study, these findings are reflected in participants’ descriptions of working under constrained conditions that they perceived as shaping how nursing assessment is carried out in their specific setting.

Interpersonal and communication challenges were also described by participants as part of their daily work environment. Nurses reported that negative patient attitudes, defensive behaviors, and reduced cooperation sometimes affected interactions during nursing assessment. Similar issues have been documented in the literature, where workplace stress, high patient volume, and limited time for engagement have been associated with strained nurse–patient communication (31). Other studies have also noted that workplace stressors may affect communication quality and contribute to misunderstandings between patients and healthcare providers (32, 33). In the present study, these experiences are understood as nurses’ accounts of how relational dynamics are encountered within a structurally constrained environment, rather than as measurable effects on patient outcomes.

Participants further described emotional and professional strain linked to sustained workload pressures, including burnout, absenteeism, and the need to adopt coping strategies such as task prioritization, delegation, and improvisation. Similar patterns have been reported in the literature, where chronic workload pressure has been associated with burnout and reduced professional well-being (34–36). Within this study context, these findings reflect nurses’ perceived experiences of working under demanding conditions, where they described adapting their practices in response to competing clinical priorities and limited resources. These accounts highlight how nurses manage care delivery in real time within the constraints of their working environment.

The Donabedian Structure–Process–Outcome framework was used as an organizing lens to interpret the findings; however, in this study it is applied as a contextual framework for understanding participants’ accounts rather than as a causal explanatory model. Nurses’ descriptions suggest that structural conditions such as staffing shortages and resource limitations are experienced as influencing how care processes, including assessment, documentation, and prioritization, are carried out in practice. These reported experiences are further linked to perceived professional and care-related challenges such as workload strain and concerns about care adequacy. Importantly, these interpretations remain grounded in participants’ accounts within a single healthcare facility and should not be understood as generalizable causal relationships.

In terms of practical implications, participants’ accounts suggest that supportive leadership and improved communication within the ward environment may be beneficial in addressing some of the challenges described. Nurses emphasized the importance of responsive management in helping them navigate workload pressures and coordinate care more effectively. These suggestions are presented as context-specific reflections from participants and may be considered in relation to broader efforts aimed at strengthening healthcare systems and workforce support, including policy priorities such as the African Union's Agenda 2063.

## 5 Study recommendations

Grounded in the findings and interpreted through the Donabedian model, the following recommendations are proposed to address structural deficiencies, strengthen care processes, and improve patient and professional outcomes.

Firstly, at the structural level, healthcare institutions and policymakers should prioritize improving nurse–patient ratios through targeted recruitment and retention strategies. Addressing staff shortages would reduce workload pressure, improve the quality of nursing assessments, and minimize burnout and absenteeism. In addition, resource allocation should be aligned with population growth and patient admission trends to ensure adequate bed capacity, infrastructure, and essential supplies. Strategic workforce planning and budget re-evaluation are essential to prevent overcrowding and ensure safe care environments.

Secondly, interventions aimed at strengthening the process of care delivery should be implemented. Healthcare facilities should provide continuous professional development focusing on effective communication skills, conflict management, and coping strategies to help nurses manage difficult patient interactions. Standardized nursing assessment protocols and supportive supervision systems should be reinforced to promote accountability and reduce the risk of incomplete assessments or documentation errors. Furthermore, clear delegation guidelines and mentoring systems should be strengthened to minimize medico-legal risks associated with task shifting under pressure.

Thirdly, to improve outcomes for nurses, institutional wellness programs should be introduced or strengthened. These may include structured rest periods, psychological support services, stress management interventions, and mechanisms for reporting workload-related concerns without fear of reprisal. Promoting nurse well-being is critical to reducing absenteeism and maintaining quality patient care.

Fourthly, patient-centered interventions should be considered. Community awareness campaigns could help address misconceptions about nurses and promote mutual respect between healthcare providers and patients. Improving patient flow systems at hospital entry points may also reduce negative patient experiences that later affect nurse–patient interactions.

Finally, future research should explore intervention-based studies that examine strategies to mitigate structural and process-related barriers identified in this study. Longitudinal research may further clarify the relationship between staffing levels, nursing processes, and patient outcomes within resource-constrained settings.

## 6 Strengths and limitations of the study

A major strength is the rich, in-depth qualitative data generated from nurses’ lived experiences, which provided detailed insight into structural and process-related challenges influencing care delivery. The use of Donabedian model further strengthened the analysis by offering a clear theoretical lens to systematically interpret the relationship between structural constraints, care processes, and outcomes. However, the study has limitations; it was conducted within a single healthcare setting, which may limit the transferability of findings to other institutions or regions. The reliance on self-reported data introduces the possibility of recall bias and social desirability bias, as participants may have underreported sensitive practices such as documentation forgery. Additionally, the study focused solely on nurses’ perspectives without incorporating views from patients, administrators, or other healthcare professionals, which may limit the comprehensiveness of the findings.

## 7 Future directions

Future studies could strengthen the credibility and depth of findings by incorporating methodological triangulation. For example, non-participant observation of nursing practice could be used to directly examine how nursing assessments and workload pressures are managed in real time, providing insight beyond self-reported experiences. In addition, reviewing patient records and clinical documentation could help verify reported challenges related to assessment completeness, documentation practices, and continuity of care. Combining these approaches would allow for cross-validation of findings, reduce reliance on self-report data, and enhance the overall trustworthiness and robustness of future research outcomes.

## 8 Conclusion

This study provides a concise, context-bound interpretation of nurses’ reported experiences of structural and organizational constraints within a single public healthcare setting. Participants described staff shortages, limited resources, overcrowding, and challenging patient interactions as part of their daily work environment, which they perceived as influencing nursing assessment and care processes. Using the Donabedian Structure–Process–Outcome framework as an interpretive lens, the findings highlight how these reported conditions are experienced as shaping clinical processes and contributing to perceived workload strain, reduced capacity for comprehensive assessment, and professional stress.

From a policy and practice perspective, the findings suggest that strengthening organizational support systems, improving staffing adequacy, and addressing resource limitations may be relevant considerations for enhancing working conditions in similar healthcare settings. However, these implications are grounded in a single-site qualitative study and should be understood as context-specific insights rather than generalizable conclusions.

## Data Availability

The datasets presented in this article are not readily available because data supporting the findings of this study are available upon reasonable request from the corresponding author. As data may contain information that jeopardizes the privacy of research participants, they are not publicly available. Requests to access the datasets should be directed to hlungwanegundo@gmail.com.
